# Beyond Conventional Imaging: Nuclear Imaging in Rheumatoid Arthritis

**DOI:** 10.3390/jcm14228127

**Published:** 2025-11-17

**Authors:** Helen Sugden, Andrea Di Matteo, Kulveer Mankia

**Affiliations:** 1 Leeds Institute of Rheumatic & Musculoskeletal Medicine, Chapel Allerton Hospital, Leeds LS7 4SA, UK; fclq345@leeds.ac.uk (H.S.);; 2 National Institute for Health and Care Research, Biomedical Research Centre, Leeds Teaching Hospitals NHS Trust, Leeds LS7 4SA, UK

**Keywords:** rheumatoid, arthritis, nuclear, scintigraphy, SPECT-CT, PET, whole-body, imaging

## Abstract

Rheumatoid arthritis (RA) is a systemic inflammatory disease characterized primarily by symmetrical small joint inflammation and damage, often accompanied by anti-cyclic citrullinated peptide (ACPA) and rheumatoid factor (RF) positivity. While conventional imaging modalities such as plain radiographs, ultrasound (US), and magnetic resonance imaging (MRI) are widely used to assess articular and some extra-articular manifestations, each presents limitations in terms of accessibility, comprehensiveness, and diagnostic scope. Nuclear imaging techniques, including positron emission tomography (PET), scintigraphy, and single-photon emission computed tomography (SPECT), offer whole-body imaging capabilities and the potential to simultaneously detect multi-system involvement, making them uniquely suited to the complex, systemic nature of RA. This review explores the current and potential roles of nuclear imaging in RA, highlighting its advantages in detecting both articular and extra-articular disease and its emerging promise as a routine tool in RA management.

## 1. Introduction

Rheumatoid arthritis (RA) is a systemic inflammatory disorder, typically presenting with symmetrical small joint polyarthritis and early morning stiffness alongside anti-cyclic citrullinated peptide (ACPA) and rheumatoid factor (RF) positivity [1]. Although extra-capsular tendon disease and articular and manifestations are the most prominent feature particularly in early disease, patients can develop extra-articular manifestations such as interstitial lung disease, rheumatoid-related myocarditis, and vasculitis, and are at higher risk of conditions including cardiovascular disease and osteoporosis [2,3,4,5,6].

Plain film radiographs (XR), ultrasound (US) and magnetic resonance imaging (MRI) are commonly used in imaging of RA joint disease [7,8]. Radiographs are helpful in the identification of bone erosions and large joint effusions, and US and MRI can confirm the presence of synovitis, tenosynovitis and subclinical synovitis, and can elucidate the degree of underlying bone damage [9]. Imaging can be particularly helpful for joints that are more difficult to assess clinically such as large joints and joints within the spine and pelvis, and it also forms an essential part of the assessment of extra-articular disease [10].

There are limitations and compromises when using any imaging technique—XR is not able to easily visualise soft tissue, and US depends on a skilled sonographer and is time-consuming, usually focused on symptomatic joints in clinical practice [11]. Similarly, obtaining MR images is a lengthy process, whole-body MRI is not widely available outside research centres and takes around 1.5 h per patient acquisition [12]. Additionally, MRI is not appropriate for all patients, for example those with claustrophobia, pacemakers, or an unsuitable body habitus, and gadolinium contrast agents can be associated with allergy or nephrogenic systemic fibrosis particularly in those with sub-optimal renal function [13].

Nuclear imaging techniques can offer rapid whole-body assessment and can visualise multi-system pathology simultaneously. It stands to reason that for multi-joint and multi-system diseases like RA, nuclear imaging techniques are uniquely exciting [14]. Here we look at how nuclear imaging can be effectively used for our RA patients and why it may form a more routine part of care for these patients in the future.

## 2. A Summary of Nuclear Imaging Techniques

Molecular imaging techniques use tracers that are introduced into the body and interact with tissues according to their biochemical properties. Nuclear imaging is a subset of molecular imaging that uses radiopharmaceuticals (radiotracers). Radiotracers are compounds tagged with a radionuclide whose emissions can be detected allowing visualization of where these compounds are sequestered, metabolised, and excreted throughout the body. Tagged compounds include analogues to natural compounds like glucose or can be engineered molecules that bind specific ligands of interest [15]. Imaging modalities using radiotracers include scintigraphy, positron emission tomography (PET), and single photon emission computed tomography (SPECT), features of each are summarised in Table 1 [15].

## 3. Identification and Quantification of Inflammation Using Nuclear Imaging

Tracer uptake in a particular area helps determine disease activity there. Tracer uptake is affected by variables including time between administration and scanning and physiological differences in uptake of tracers within different tissues. These factors can be taken into account and imaging data can be analysed using semi-quantitative assessments to evaluate disease activity in a specific joint or globally [19].

To analyse nuclear medicine images, a common technique is to define a volume of interest (VOI) if 3D and a region of interest (ROI) if 2D. In rheumatology, a volume of interest might be the knee joint, for example. Areas can be mapped using fixed sized regions, manual mapping, or algorithms. ‘Standardised uptake value’ (SUV) is the most commonly used uptake measure and refers to tracer uptake for each pixel or voxel (3D pixel) within the VOI or ROI compared to the average uptake through the whole body. A high SUV infers that that area has a higher concentration of radioactivity than we would expect if the tracer had distributed equally throughout the body. SUVmax refers to the pixel or voxel within the VOI or ROI with the highest SUV [20,21,22,23].

Scintigraphy was the first nuclear imaging technique used to quantify RA disease activity, it is highly sensitive for joint inflammation and outperforms clinical examination in the identification of histologically confirmed synovitis [24,25,26]. Over decades and with various tracers, scintigraphy has been shown to correlate with markers of disease activity; tender and swollen joint counts, biochemical markers such as CRP and erythrocyte sedimentation rate (ESR), and composite scores DAS-28-CRP and DAS-28-ESR [27,28,29,30,31,32,33,34,35]. The same is true for SPECT imaging [15,36,37,38,39].

PET was first used in RA assessment in the 1990s. Since then, several studies have confirmed correlation between PET activity and clinical joint swelling and tenderness, but not with patient reported outcomes indicating additional factors above joint inflammation affect patient experience [40,41,42,43,44,45,46,47,48,49,50]. Additionally, 18F-FDG PET has been shown to demonstrate uptake in tendon sheaths and bursae [50].

In some studies, 18F-FDG PET was less successful in low disease activity groups [51,52]. This may be due to FDG imaging having lower specificity for joint inflammation meaning that joints with low level inflammatory change may be missed [53,54,55].

Several studies using PET to assess treatment responses in RA have shown that changes in tracer uptake reflects improvement in disease control, demonstrating its utility in quantification of disease activity [40,41,44,49,50,56,57,58,59,60,61,62,63,64,65,66]. PET is useful in assessing larger joints, the atlanto-axial joint, and the sacro-iliac joints, where it is more difficult to differentiate non-inflammatory and inflammatory pain clinically [67,68,69,70]. Notably, there are lower rates of intra-observer variability when evaluating disease using PET imaging compared to clinical examination [41].

Lee et al. developed a composite score assessing disease activity taking in account PET28 counts (i.e., PET positivity or negativity in joints involved in DAS28 calculations). This correlates well with DAS28-ESR [43]. They completed similar work using bone scintigraphy, and again demonstrated good correlation of BSS28 (bone scintigraphy score) with TJC28, SJC28, DAS28-ESR, and PET28 counts. Using BSS28 in addition to ESR and patient global assessment, the team created a composite score that strongly correlated with DAS28-ESR. Given the lower cost and wider availability of scintigraphy compared to PET, this has clear benefit [29].

## 4. How Do Nuclear Imaging Techniques Compare to Conventional Imaging?

Scintigraphy is comparable to conventional MRI and US imaging in identifying inflamed joints in RA [24,25,71,72,73,74]. 18F-FDG and 11C-choline PET tracer uptake strongly correlates with MRI synovitis with areas of highest uptake corresponding with synovial thickening on MRI [48,49,75]. 18F-FDG PET has a higher detection rate for inflammation within the ischial tuberosities and sacroiliac joints compared to MRI in RA, polymyalgia rheumatica, and axial spondyloarthropathy patients [69].

**Figure 1:**
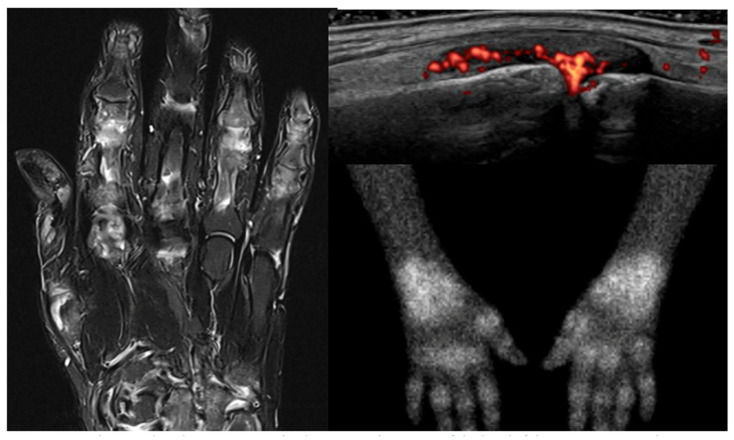
MRI, ultrasound, and 99mTc-Maraciclatide scintigraphy images of the hand(s) of the same patient with rheumatoid arthritis.

Scintigraphy produces rapid whole-body images with ~30 min image acquisition time, compared to 90 min required for whole-body or robust joint set images with MRI and US [12,14]. However, a delay is required between tracer injection and image acquisition to allow for adequate tracer distribution, this delay varies between tracers. Compared to an MRI scanner, the scintigraphy scanner is more open and easier to tolerate. Articular views produced by scintigraphy are easy to interpret, and analysis of these images is rapid and can be done using automated algorithms, though they do not provide the same level of anatomical detail as can be demonstrated with MRI and US. Figure 1 shows examples of each.

PET is considerably more expensive compared to scintigraphy, MRI, and US, and is less widely available, though shows excellent utility in identification of joint inflammation. Beckers et al. scanned a limited joint set in 21 RA patients using 18F-FDG PET and ultrasonography. Of the joints assessed 75% were swollen, 63% were PET positive, and 56% were USS positive, indicating that PET scanning was able to detect more clinically swollen joints compared to ultrasound. Joints were PET positive in 96% of joints where ultrasound power Doppler was present, and 83% of ultrasound positive joints with hypoechoic or anechoic areas within the joint space but without power doppler. There was also good correlation between SUV levels on PET scanning and synovial thickness on ultrasound in all joints except the first MTP [41].

18F-FDG and 11C-choline PET tracer uptake strongly correlates with MRI synovitis and clinical swelling, and areas of highest uptake correspond with synovial thickening on MRI [48,49,75]. 18F-FDG PET has been shown to have a higher detection rate for inflammation within ischial tuberosities and sacroiliac joints compared to MRI in RA, polymyalgia rheumatica, and axial spondyloarthropathy patients [69].

Limitations of nuclear imaging techniques include radiation exposure, potential for tracer allergy or extravasation, and logistical challenges such as varying time scales required between injection and image acquisition depending on tracer used, and advice to avoid young children and pregnant people for several hours following tracer injection and abstain from having blood tests for a few days owing to radioactivity of bodily fluids. It is important to note the difference in radiation exposure between nuclear imaging scan types, with exposure from PET-CT scanning being five times higher than scintigraphy, this is an important consideration particularly in regard to serial scanning [16,18].

Currently there are no contemporaneous cost effectiveness analyses for the use of nuclear imaging techniques for inflammatory arthritis, and this is certainly an area that requires further investigation.

## 5. Novel Tracers

As suggested in Table 2, one of the benefits of nuclear techniques in RA imaging is the scope to develop radiotracers targeted towards specific cells and pathways involved in the inflammatory process, this is a major area of development and has the potential to improve specificity of these techniques for rheumatoid-related inflammation. To date, tracers targeting fibroblasts, macrophages, and activated vascular endothelium have all been used experimentally and are discussed below.

### 5.1. Fibroblast-Targeting Tracers

Fibroblasts are key players in synovial inflammation development, depletion of fibroblast activation protein (FAP)-expressing fibroblasts is associated with lower levels of arthritis [76,77]. PET uptake of tracers such as 18F-FAPI, 68Ga-FAPI which bind FAP correlates with DAS28-CRP scores [78,79,80]. Interestingly, a lack of uptake of 68Ga-FAPI within clinically inflamed or swollen joints on PET/CT was associated with a lower likelihood of response to csDMARD and bDMARD treatment. This could be due to fibroblast imaging differentiating between tenderness and swelling due to previous damage as opposed to active inflammation [80].

FAP imaging is able to differentiate between active inflammation and fibrosis in IgG4 related disease and has also been able to demonstrate fibrosis in interstitial lung disease secondary to systemic sclerosis [81,82]. The potential to image both articular and lung disease simultaneously has huge benefits in RA.

### 5.2. Macrophage-Targeting Tracers

Macrophages are polarised towards an M1 or M2 phenotype; the M1 phenotype is pro-inflammatory where the M2 phenotype is anti-inflammatory [83]. High numbers of macrophages within the RA synovium are associated with more severe joint damage and higher disease activity scores, and although numbers of both M1 and M2 macrophages are raised in active rheumatoid joints, there is a higher proportion of M1-types [84,85]. M2 macrophages predominate in healthy joints, and the joints of RA patients in remission [83,86,87]. Macrophage imaging has therefore been suggested as a potentially more specific tracer for joint inflammation compared to 18F-FDG [88].

Tracers such as (R)-[11C]PK11195, 18F-DPA-714, and 18F-DPA-713 bind to peripheral benzodiazepine receptors, types of translocator proteins (TSPOs), predominantly found on macrophages and monocytes. Imaging using (R)-[11C]PK11195 is helpful in predicting future flare of RA in patients in remission as well as in assessing disease activity [53,54,55,89,90].

Additionally, folate receptor beta, a plasma membrane protein strongly expressed on M2 macrophages is a potential macrophage tracer target [91]. 18F-fluoro-PEG-folate uptake on PET/CT correlates well with clinically active joints, but with lower background uptake compared to 18F-FDG PET/CT meaning that subtle arthritis was more easily visualised [92]. 99mTc-EC20—folic acid conjugated with 99mTc—has been utilised to image the folate receptor though using scintigraphy rather than PET and showed 47% sensitivity and 85% specificity for joint swelling identified on clinical examination, uptake correlated well with ESR and CRP, and subclinical inflammation was detected in 180 joints from 40 RA patients [93].

### 5.3. Activated Vascular Endothelium-Targeted Tracers

Vascular endothelial dysfunction is seen in both large and small vessels in RA, it is implicated in the increased prevalence of atherosclerotic disease in RA cohorts, and in synovitis-associated neo-angiogenesis respectively [94,95].

**Figure 2:**
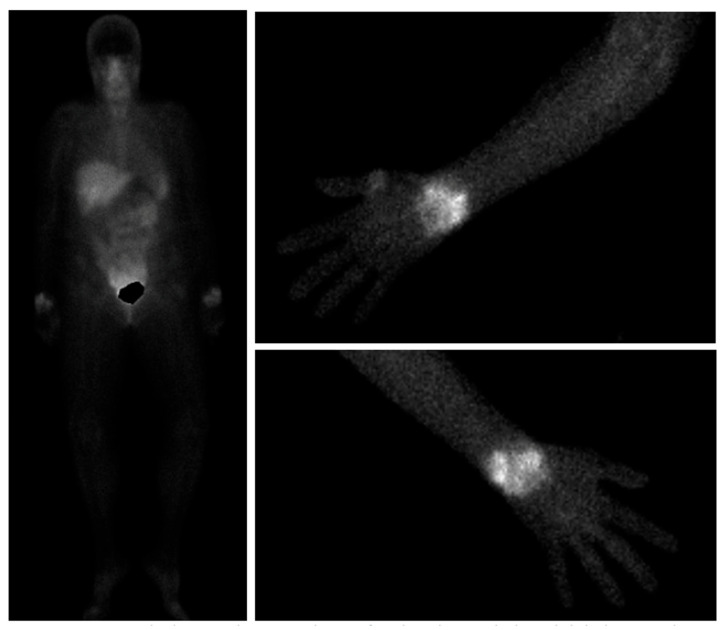
99mTc-Maraciclatide images showing visualisation of uptake in the wrists both on whole-body views and dedicated upper limb views in a patient with rheumatoid arthritis.

Integrin aVB3 is involved in neo angiogenesis in RA, macrophage-dependent inflammation, and bone homeostasis [96]. 99mTc-Maraciclatide binds this integrin with high affinity, and scintigraphy imaging uptake correlated strongly with ultrasonographic and clinical findings in 100 RA patients with low, moderate, and high disease activity [97]. 99mTc-Maraciatide scintigraphy findings strongly correlate with ultrasound findings in RA, an example of Maraciclatide scintigraphy imaging can be seen below in Figure 2 [97]. Further studies using this tracer are ongoing, comparing it to MRI and ultrasonography in RA and psoriatic arthritis, assessing its utility in prediction of successful TNF inhibitor tapering in RA, and also in identification of interstitial lung disease [98,99,100].

### 5.4. Radiolabelled Biologics

Biologic medications have been a hugely important breakthrough in RA treatment. By using radioactive isotopes to tag biologic medications to create tracers such as 123I-IL-1ra, 99mTc-human-anti-TNF-mAb, 99mTc-labelled certolizumab pegol, 99mTc-infliximab, and 124I-labelled-rituximab, we can show where these drugs are concentrated within the body [73,101,102,103,104,105,106]. This is beneficial in drug selection. It has been demonstrated that increased uptake of 99mTc-lableled certolizumab-pegol within a joint is correlated with resolution of pain following certolizumab treatment, compared to in painful joints without uptake [101]. Additionally, 99mTc-infliximab can be useful in identifying persistent mono-arthritis in RA that would be responsive to intra-articular infliximab injection [104]. There is clear potential here in pre-determining biologic treatment success without exposing patients to potential adverse effects of these medications and allowing patient-specific drug selection.

## 6. Subclinical Synovitis

Subclinical synovitis refers to inflammation that is not detectable on physical examination. At the joint level it has been shown to be associated with progressive bone erosion and can help predict development of RA in at-risk individuals [107,108,109,110,111,112,113,114].

Gent et al. reviewed MCPs, PIPJs, and wrists of patients with RA in clinical remission using a macrophage-targeting tracer, 11C-(R)-PK11195; the definition of remission was not specified. They demonstrated that subclinical inflammation was seen in 55% of patients (*n* = 16) and 8% of imaged joints (*n* = 50) on 11C-(R)-PK11195 PET/CT [89,115].

Verweij et al. utilised the same technique in active early arthritis patients pre-treatment [116]. 35 patients had 11C-(R)-PK11195 PET/CT scanning and DAS44 scoring. PET positivity or negativity corresponded with presence or absence of tenderness or swelling in 74 and 75% of joints respectively. They found that 83 out of 1400 joints (5.9%) assessed were PK11195 PET/CT positive but clinically not swollen. 12.1% of clinically non-swollen large joints (shoulders, wrists, knees, and ankles) were PET positive. This may indicate a benefit in the use of PET imaging to assess subclinical disease in larger joints, perhaps indicating low volume inflammation that is clinically imperceptible [116].

99mTc-pyrophosphate scintigraphy scanning has shown that radionuclide activity can precede synovitis and or radiographic changes at the joint level [117]. Subclinical disease detected by scintigraphy may be predictive of flare, with evidence showing that a high proportion of joints found to be sub-clinically active on scintigraphy scanning become clinically synovitic with follow up [28].

## 7. Differentiation Between RA and Other Types of Arthritis

In some cases, it is difficult to differentiate types of arthritis as well as active inflammation versus previous damage. This is an important factor when considering treatment escalation, particularly in the polyrefractory and EULAR described ‘difficult to treat’ RA cohorts, where higher disease activity scores may be driven by previous damage rather than ongoing active inflammation [118,119]. Radiotracer uptake on scintigraphy or PET/CT scanning is attenuated or absent in non-inflammatory joints, including joints with erosions, and is also helpful in the assessment of larger joints, where it can be more difficult to identify swelling and synovitis clinically [27,67].

Tender or painful joints in patients with fibromyalgia and osteoarthritic joints without secondary synovitis do not show tracer uptake on 18F-FDG PET scanning, though as with other imaging techniques, uptake in a synovitic osteoarthritic joint is indistinguishable from a synovitic rheumatoid joint, particularly in PIP joints [120]. Multi-pinhole single photon emission computer tomography (MPH-SPECT) has shown a difference in the appearance of uptake, with a higher proportion of eccentric uptake in inflammatory osteoarthritic joints and central uptake in inflammatory rheumatoid joints [121].

Synovitic joints in seronegative spondyloarthropathy patients differ in 18F-FDG PET appearance compared to synovitic RA joints, which are more homogenous, higher grade, and symmetrical [122]. RA patients can also have increased uptake in lymph nodes surrounding inflamed areas, and this phenomenon is not seen in spondyloarthropathy patients [122]. Uptake in entheses, spine, and sacroiliac joints have also been successfully demonstrated using 18F-FDG PET imaging in patients with seronegative spondyloarthropathies, including psoriatic arthritis, where nail matrix abnormalities can also be demonstrated [47,122,123,124,125].

**Figure 3:**
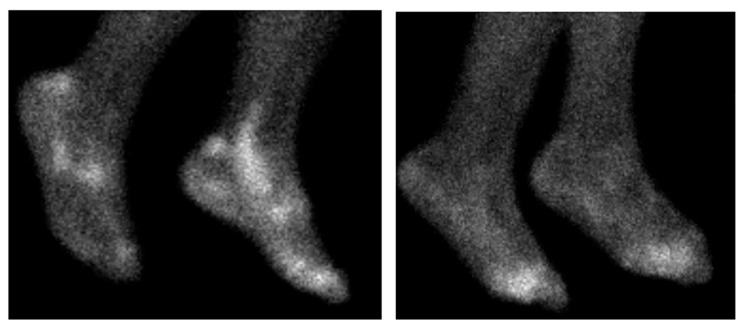
99mTc-Maraciclatide scintigraphy scans of the feet. The left showing a patient with psoriatic arthritis, with characteristic uptake within the achilles enthesis and peroneal tendons. The right showing a RA patient with metatarsophalangeal joint uptake.

For all nuclear imaging modalities, tracer uptake generally reflects the classical distribution of disease as demonstrated in Figure 3 [120]. When comparing uptake patterns in RA, spondyloarthritis, and polymyalgia rheumatica patients higher SUVmax levels are seen at sacroiliac joints in spondyloarthritis patients, and higher levels at greater trochanters in polymyalgia rheumatica patients [69].

One group has used 18F-FDG PET imaging to develop a composite score encorporating SUVmax, metabolically active volume, and total lesional glycolysis, to differentiate between rheumatoid (*n* = 18) and non-RA (*n* = 17) [126]. The non-RA group comprised patients with undifferentiated arthritis, SAPHO syndrome, IgG4 arthritis, and psoriatic arthritis. Scores were significantly higher in the RA group. The study was limited by low participant numbers, and there was no comparison of disease activity between groups on a clinical level [126].

These findings highlight the potential of nuclear imaging not only in detecting inflammatory activity but also in distinguishing RA from other rheumatological conditions, supporting its growing role in diagnostic refinement and personalised treatment strategies.

## 8. Prediction of Disease Course

RA is a heterogenous condition, some patients obtain excellent early disease control with csDMARD monotherapy, where others experience prolonged inflammation and become ‘difficult to treat’ or ‘poly-refractory’ [119]. Smoking is positively associated with development of polyrefractory RA [118]. Other variables including disease duration, DAS28-CRP, and tender and swollen joint counts were not found to be predictive [118]. Nuclear imaging techniques are helpful in predicting flare or development of erosions, but to our knowledge, there have been no studies looking at whether nuclear imaging techniques can predict development of a more difficult to manage rheumatoid phenotype [27,127].

Thirteen patients with active RA were prospectively assessed by Möttönen et al. in 1988 [27]. They had 99mTc-methylenediphosphonate scintigraphy scanning at baseline, 6 and 12 months alongside clinical assessment of tenderness. X-rays were also completed at 0, 6, 12, 18, and 24 months. All 6/6 hand joints and 21/22 foot joints that developed radiographic erosions at 12 months were scintigraphically active at baseline. In the feet, positive scintigraphy at baseline had a sensitivity of 86% and specificity of 95% in prediction of erosion development at 12 months, this compared to clinical activity score of 3 or higher, which had a sensitivity of 86% but a low specificity of 46%. Clinical activity scores considered tenderness within a joint only and graded this on a scale of 0–3. Similar sensitivity was seen using 99mTc-IgG scintigraphy, though with inferior specificity [33].

Using PET, higher levels of 18F-FDG uptake in large joints is highly correlated with later radiographic erosions, in that joint. Additionally, higher SUVmax levels pre-treatment are a significant predictor for progressive bone erosion after 3 years [62,128,129].

Elzinga et al. used 18F-FDG PET/CT at baseline and after 2 weeks of treatment with infliximab and followed patients for 22 weeks. Changes in SUVmean between baseline and 2 weeks correlated significantly with DAS28 outcomes at 14 and 22 weeks. This indicates that very early treatment response is a predictor of outcomes, if we can accurately determine within just a few weeks which patients will and will not respond well to treatment, it may allow for accelerated decision making regarding medications [130].

Regarding prediction of flares, a single study using 11C-PK11195 PET has shown promise in predicting development of flare for patients in remission or with low disease activity within the next 3 years with those who flare having higher cumulative PET scores whilst in remission [53,89].

Remission is the goal for RA patients, and once in stable remission, minimising patient exposure to anti-rheumatic drugs by tapering these medications is preferable [127,131,132,133]. There are several factors that have been found predictive of flare versus successful tapering and cessation of biological therapy, factors include lower disease activity at baseline, absence of imaging detected inflammation, longer remission duration, better patient-reported outcomes, and lower CRP and ESR levels at baseline [134,135,136,137].

Bouman et al. assessed whether inflammation detected using FDG-PET imaging could have utility in prediction of successful TNFi tapering and cessation. Of 79 patients enrolled, 47% of patients (*n* = 37) had successfully reduced TNFi doses at 18 months, and 20% (*n* = 16) had successfully discontinued. SUVmax and SUVmean at baseline between those who weren’t able to taper at all, those who were able to taper partially, and those who were able to taper to cessation were not significantly different and the team were not able to demonstrate that 18F-FDG PET/CT scores were able to assist in prediction of successful tapering or discontinuation of TNFis [52]. This is reflective of earlier remission work using ultrasound imaging [135,136]. This could indicate that subclinical joint inflammation is predictive of flare within the short-term only, serial imaging during tapering may be beneficial in elucidating this further.

A drug tapering study using scintigraphic imaging and a 99m-Tc labelled molecule that binds with high affinity to avB3 integrin expressed on angiogenic blood vessels is in progress [98]. Further work in this area is required.

## 9. Prediction of Future Arthritis Development in at Risk Individuals

The definition of ‘at risk’ of RA has not been universally defined and can take the form of ACPA and or RF positivity alongside musculoskeletal symptoms including early morning stiffness and arthralgia, particularly when involving small joints of the hands, others define undifferentiated arthritis as an at risk population [110].

Conventional imaging findings using MRI and ultrasound correlate with future development of inflammatory arthritis in these at risk cohorts [111,138,139,140,141]. Nuclear imaging techniques have also been shown to have predictive value; 11C-PK11195 tracer uptake on PET imaging is associated with arthritis development within 2 years in at risk ACPA positive patients [142]. Gent et al. assessed 29 ACPA positive patients with arthralgia with clinical examination and 11C-PK11195 PET of the hands and wrists. Four patients had tracer uptake in at least 1 joint and all 4 of these patients progressed to clinical arthritis by 24 months. Those without tracer uptake within the joints did not progress within the follow up period, this prognostic information could be extremely valuable in reassuring patients by identifying patients at very low risk of progression despite other risk factors [142].

Nuclear imaging techniques have also been shown to be helpful in more heterogenous cohorts including both at risk with arthralgia and undifferentiated arthritis patients. Duer et al. compared contrast enhanced MRI of the wrists and metacarpophalangeal joints of the most symptomatic hand and whole-body bone scintigraphy in 41 patients with undifferentiated arthritis. Patients were tentatively labelled as RA if MRI and scintigraphic images showed subclinical joint inflammation in the hands that reflected a classical rheumatoid arthritis distribution (*n* = 13). Within 2 years, 11/13 of these patients fulfilled the 2010 ACR classification criteria for RA diagnosis. The remaining 28 undifferentiated arthritis patients whose scintigraphy imaging was not in keeping with RA did not progress to RA at 2 years [143].

A cohort of 51 patients with symptoms including pain, swelling, or <30 min morning stiffness who did not meet criteria for RA were reviewed by Ozgul et al., again this cohort included both undifferentiated arthritis and at risk arthralgia patients [71]. Participants had ultrasonography and whole-body bone scintigraphy scanning at baseline and were followed up for 2 years. Thirty-three patients met 2010 ACR classification criteria for RA by 2 years. Although both imaging modalities correlated with development of RA, there was a higher predictive value for ultrasound detected synovitis at baseline and progression compared to bone scintigraphy [24].

De Bois et al. identified 47 patients with arthralgia in multiple joints and no clinical evidence of synovitis alongside at least one of the following features: joint stiffness, limited joint motion, tenderness on palpation. Patients received 99mTc-IgG scintigraphy scanning at baseline and were followed up for 12 months. Over the follow up period, 8 patients progressed to RA and 7 of these patients had tracer uptake within their joints at baseline. Of the remaining patients who did not progress, only 1 patient had tracer uptake in any joint [144].

Whole body imaging in the context of the pre-RA disease state where symptoms are less well defined and localised offers potential for early detection detection and opportunity to intervene to prevent or delay development of RA. There is significant scope to use nuclear medicine techniques to help identify those at the highest risk (Table 3).

## 10. Extra-Articular Disease

Extra-articular manifestations such as myocarditis, interstitial lung disease, and vasculitis, affect around 40% of RA patients at some point during the disease course [145]. In addition, patients with RA are at higher risk of conditions including cardiovascular disease and osteoporosis [146].

Nuclear imaging techniques can demonstrate inflammatory pathology at the whole-body level which is helpful in assessing whole-body disease burden. 18F-FDG PET CT can visualise synovial and vascular inflammation simultaneously [65], vascular inflammation being an integral part of atherosclerotic disease as well as rheumatoid vasculitis, rheumatoid nodules, and associated thyroiditis [122].

PET can be used to assess coronary blood flow and subclinical atherosclerosis [147,148]. The TARGET study assessed RA patients who had failed methotrexate monotherapy and who were randomised to receive either TNFi therapy or triple therapy with methotrexate, sulfasalazine, and hydroxychloroquine. Participants had 18F-FDG PET scanning at baseline and at study completion after 24 weeks, FDG uptake was assessed in the aorta and carotid arteries. The results demonstrated significant reduction in arterial uptake between baseline and follow-up, independent of treatment regime [149]. The study did not report on differing uptake at the articular level, but extrapolating from other studies which have shown reduction in 18F-FDG uptake at the joints with treatment, we can surmise that 18F-FDG PET imaging can be used to visualise multi-organ inflammation simultaneously [58,60,62,63,64,65]. Although 18F-FDG PET is useful in imaging vascular inflammation, it is not able to differentiate blood vessel calcification, an important factor in the development of cardiovascular disease, other PET tracers such as 18F-Na-F are more successful in imaging vascular calcification [150].

Nuclear imaging also has a role in the evaluation of lung fibrosis which can be seen more frequently in RA patients, and although there is literature describing 18F-FDG PET activity in the lungs in scleroderma patients, this has not yet been assessed in RA patients to our knowledge [151,152].

In any case, caution must be exercised when using whole-body imaging due to the potential for promoting over-investigation of benign lesions when up to 50% of RA patients having 18F-FDG PET CT can be found to have extra-articular abnormalities with only a small proportion of these being clinically concerning on further investigation [153]. Nuclear imaging techniques are not able to differentiate easily between rheumatoid manifestations such as rheumatoid nodules and malignancy and so could increase investigative and diagnostic procedures unnecessarily [154].

## 11. Next Steps for Nuclear Imaging in RA

Radiopharmaceuticals tailored to detect disease targets fundamental to the pathophysiology of RA clearly have potential in sensitively and specifically imaging RA-related inflammation. Further exploration of their utility in prediction, diagnosis, and prognosis in RA is required. In particular, the development of tracers that can identify RA-related inflammation in multiple organ systems offers an exciting opportunity to evaluate and manage RA, not as a solely articular disease, but a systemic inflammatory disorder. Nuclear imaging could be integral in assessing response of extra-articular disease to treatments, and subsequently in optimising of preventing and managing inflammatory sequelae and comorbidity, though the associated radiation exposure must be considered. Patients with established systemic disease have the highest levels of healthcare burden, improving detection and prevention is extremely valuable both financially and at the patient level.

Within nano-therapeutics, radiopharmaceutical tracers that can be specifically concentrated in areas of active inflammation via chemically engineered binding motifs would allow optimisation of localised drug delivery to areas of high disease burden, potentially reducing the likelihood of adverse effects and improving drug efficacy. Nuclear imaging can visually demonstrate the success of these binding motifs in promoting sequestration of a labelled molecule in areas of inflammation [155,156].

On a practical level, images produced by nuclear techniques are easy to interpret for both non-specialist physicians and lay-people alike and so can form a uniquely important part of a clinician-patient interaction. Being able to clearly and easily see inflammation within the joints on screen can motivate treatment adherence, where being able to clearly see a lack of inflammation for those patients in remission may bolster confidence in medication tapering. Excitingly, artificial intelligence can be used to rapidly interpret scintigraphy images with similar performance to a clinician. Thus, there is potential both for rapid whole-body imaging with automatic assessment of joint inflammation [157].

## 12. Conclusions

Nuclear imaging is comparable to both MRI and ultrasonography in demonstrating articular inflammation in rheumatoid arthritis but has other wide-ranging advantages.

Further research is required to assess the capacity of newer, targeted radiotracers to predict RA development in patients deemed at risk, as well as in prediction of successful drug tapering. A robust comparison between scintigraphy and PET/CT would be beneficial to determine whether there are any significant benefits in using PET given the increased cost and radiation exposure. Additionally, whether nuclear imaging can demonstrate extra-articular and articular inflammation simultaneously requires further exploration, as this offers the potential to show a true reflection of whole-body inflammatory burden in RA and a chance to intervene to prevent extra-articular complications of RA at an early stage.

## Figures and Tables

**Table 1:** An overview of features of different nuclear imaging techniques.

Scan Type	Scintigraphy	SPECT	PET CT
Isotope	Gamma emitting isotopes	Gamma emitting isotopes	Positron emitting isotopes
Radiotracer	Radioisotope with additions (e.g., binding motifs, molecular linkages, chelators)	Radioisotope with additions (e.g., binding motifs, molecular linkages, chelators)	Radio-labelled analogue to natural compound or Radioisotope with additions (e.g., binding motifs, molecular linkages, chelators)
Radiation dose (whole body)	~4 mSV (Equivalent to 20 chest X-rays) [16]	~7 mSv (Equivalent to 35 chest X-rays) [17]	~20 mSv for PET CT (Equivalent to 100 chest X-rays) [18]
Image dimension	2D	3D	3D
Resolution	Low resolution	Medium to high resolution	High resolution
Acquisition time	Long acquisition time	Medium to long acquisition time	Short acquisition time
Availability	Widely available	Widely available	More limited availability
Tracer cost	Low cost	Low cost	High cost

Acronyms. SPECT: single photon emission computed tomography, PET CT: positron emission computed tomography, mSv: millisieverts, 2D: 2 dimensional, 3D: 3 dimensional.

**Table 2:** Comparison of some of the pros and cons of MRI, USS, and scintigraphy/SPECT/PET imaging.

MRI	USS	Scintigraphy/SPECT/PET
Long image acquisition times or symptomatic joint focused imaging	Long image acquisition times or symptomatic joint focused imaging	Rapid whole-body image acquisition times, but variable delays between tracer injection and scanning
Excellent anatomical detail including views of deeper structures	Good anatomical detail, poorer visualisation of deeper joints and structures	Crude anatomical detail but good views of deeper structures and opportunity to visualise metabolic processes via targeted tracers
No radiation exposure	No radiation exposure	Radiation exposure
Contrast agent used in some cases	No contrast agent	Radiotracer used
Images require specialist interpretation	Operator dependent and images require specialist interpretation	Opportunity for immediate algorithmic image interpretation including quantification of level of inflammation
Not suitable for claustrophobic patients, or those with certain implants	Suitable for all	Not suitable for pregnant patients or young children

**Table 3:** Summary of findings surrounding topic of “at-risk” individuals and nuclear imaging.

Author	Population	Outcome
Gent et al., 2012 [142]	ACPA + ve arthralgia patients (*n* = 29)	11C-(R)-PK11195 PET CT joint uptake associated with development of arthritis within 2 years
Duer et al., 2008 [143]	Unclassified arthritis > 6 months patients (*n* = 41)	Subclinical synovitis pattern demonstrated by whole-body bone scintigraphy uptake predictive of RA vs. non-RA progression within 2 years
De Bois et al., 1996 [144]	Arthralgia patients (*n* = 52)	99Tc-IgG scintigraphy had an 88% sensitivity, 97% specificity, and 88% positive predictive value for RA development within 1 year

## Abbreviations

The following abbreviations are used in this manuscript:

RA	Rheumatoid arthritis
ACPA	Anti-cyclic citrullinated peptide
RF	Rheumatoid factor
US	Ultrasound
MRI	Magnetic resonance imaging
PET	Positron emission tomography
SPECT	Single-photon emission computed tomography
XR	Plain film radiograph
USS	Ultrasound scan
VOI	Volume of interest
ROI	Region of interest
SUV	Standardised uptake value
CRP	C-reactive protein
ESR	Erythrocyte sedimentation rate
DAS	Disease activity score
FDG	Fluorodeoxyglucose
BSS	Bone scintigraphy score
TJC	Tender joint count
SJC	Swollen joint count
FAP	Fibroblast activation protein
csDMARD	Conventional synthetic disease modifying drug
bDMARD	Biologic disease modifying drug
TSPO	Translocator protein
TNF	Tumour necrosis factor
MCP	Metacarpophalangeal
MTP	Metatarsophalangeal
PIPJ	Proximal interphalangeal joint
EULAR	European Alliance of Associations for Rheumatology
MPH	Multi-pinhole
SAPHO	Synovitis, acne, pustulosis, hyperostosis, osteitis
IgG	Immunoglobulin G
CT	Computed tomography
TNFi	Tumour necrosis factor inhibitor
ACR	American College of Rheumatology
